# Decreased Expression of Retinoblastoma Protein-Interacting Zinc-Finger Gene 1 Is Correlated With Poor Survival and Aggressiveness of Cervical Cancer Patients

**DOI:** 10.3389/fonc.2019.01396

**Published:** 2019-12-12

**Authors:** Shanshan Yang, Tianbo Liu, Haiyan Cheng, Zhao Wang, Yue Feng, Jiazhuo Yan, Sijia Liu, Yunyan Zhang

**Affiliations:** ^1^Department of Gynecological Radiotherapy, Harbin Medical University Cancer Hospital, Harbin, China; ^2^Department of Gynecology, Harbin Medical University Cancer Hospital, Harbin, China

**Keywords:** cervical cancer, RIZ1 expression, prognosis, proliferation, migration, invasion

## Abstract

**Background:** Retinoblastoma protein-interacting zinc finger gene 1 (RIZ1) is a tumor suppressor deregulated in several human cancers. We aim to (1) explore RIZ1 expression in FIGO stages I–II cervical cancer tissues and its association with the clinical outcome of cervical cancer patients, (2) the role of RIZ1 in proliferation, apoptosis, migration, and invasion in cervical cancer cells.

**Methods:** The expression of RIZ1 in 268 cervical cancer tissues and 30 paired adjacent non-tumor tissues were assessed by immunohistochemistry. We also examined RIZ1 at mRNA and protein level in 20 paired fresh frozen cervical cancer tissues and the adjacent non-tumor tissue using real-time PCR and western blot. We then examined proliferation, apoptosis, migration, and invasion in two human cervical cancer cells, HeLa and SiHa, with overexpression of RIZ1.

**Results:** RIZ1 expression generally decreased in cervical cancer tissues. Decreased RIZ1 expression was significantly correlated with advanced FIGO stage (*P* = 0.005), deep stromal invasion (*P* = 0.001), lymphovascular space involvement (*P* = 0.041), pelvic lymph node metastasis (*P* = 0.005), and postoperative recurrence (*P* = 0.002). Kaplan-Meier analysis demonstrated that patients with low RIZ1 expression had shorter overall survival (OS) and disease-free survival (DFS) than those with high RIZ1 expression. Multivariate analysis showed that RIZ1 was an independent prognostic factor for DFS (HR = 2.184, 95% CI 1.365–3.496, *P* = 0.001) and OS (HR = 1.899, 95% CI 1.112–3.241, *P* = 0.019). *In vitro* analysis demonstrated that overexpression of RIZ1 inhibited cell proliferation, migration, and invasion, but promoted apoptosis in HeLa and SiHa cells.

**Conclusion:** Down-regulation of RIZ1 may contribute to tumor migration, invasiveness, and poor survival of cervical cancer patients. RIZ1 may be a prognostic biomarker for cervical cancer patients.

## Introduction

Cervical cancer is the fourth cause of cancer-related mortality in women worldwide (1). The incidence of cervical cancer continuously increases, particularly in developing countries. Each year, there are about 500,000 new cases and 275,000 deaths of cervical cancer worldwide (2). In China, the estimated number of new cases and deaths from cervical cancer were 130,000 and 50,000 each year (3). Despite the advance in surgery, chemotherapy and radiotherapy, the mortality rate of cervical cancer remains high mainly because of tumor recurrence (4, 5). Therefore, identification of potential markers and elucidation of the molecular mechanisms underlying development and progression of cervical cancer is of high importance to develop new therapeutic strategies.

Retinoblastoma protein-interacting zinc finger gene (*RIZ*) is located within tumor suppressor gene clusters on chromosome 1q36 and was isolated through functional screening for Rb-binding proteins (6). *RIZ* gene encodes two proteins, RIZ1 and RIZ2. RIZ1 contains a positive regulatory (positive regulatory domain I binding factor 1 and RIZ) domain, but RIZ2 lacks this domain (7). RIZ1 displays strong tumor suppressive activities, and loss-of-function mutation or deletion, and altered DNA methylation of RIZ1 have been associated with the progression of various cancers, such as ovarian carcinoma, lymphomas, colorectal cancer and glioma (8–11). Moreover, RIZ1 is involved in carcinogenesis-related processes (i.e., cell cycle, differentiation and apoptosis) in various cancers, such as breast, liver, and colon cancers (10, 12–14). In our previous study, we found significantly reduced or even loss of RIZ1 expression in cervical cancer tissues compared with normal cervical tissues, and the decreased expression is caused at least partially by aberrant DNA methylation of the RIZ1 promoter (15). Taken together, RIZ1 could be a valuable diagnosis and therapeutic target for cervical cancer. However, to date, there is no study examining the relationship between the expression level of RIZ1 with clinicopathological characteristics and clinical outcomes in cervical cancer patients.

In this study, we retrospectively examined the expression of RIZ1 in 268 cervical cancer patients, and found that RIZ1 expression is negatively correlated with clinicopathological characteristics as well as clinical outcomes, including overall survival (OS) and disease-free survival (DFS). In two cervical cancer cells with overexpression of RIZ1, HeLa, and SiHa, we found overexpression of RIZ1 inhibits proliferation, migration and invasion, and induces cell arrest and apoptosis. In summary, we, for the first time, provided clinical and experimental evidence that RIZ1 may be an independent prognostic factor for cervical cancer.

## Materials and Methods

### Patients and Specimens

Two hundred and sixty eight cervical cancer patients who underwent surgery at the Department of Gynecology, the Affiliated Tumor Hospital of Harbin Medical University (Harbin, China) between January 1st, 2008 and December 31st, 2010 were recruited in our study. The inclusion criteria included: (1) cervical cancer at stages I-II according to International Federation of Gynecology and Obstetrics (FIGO) staging system 2009; (2) pathologically confirmed with squamous cell carcinoma or adenocarcinoma; and (3) with complete clinical data; and (4) no previous chemotherapy, radiotherapy, or immunotherapy before surgery. The exclusion criteria included: (1) patients with history of other cancers; (2) patients received chemotherapy, radiotherapy, or immunotherapy before the surgery; (3) patients with paraaortic lymph node metastasis; and (4) patients without clear follow-up results. All patients underwent surgical resection of radical hysterectomy and pelvic and/or paraaortic lymphadenectomy. Postoperative radiotherapy was given to patients with high-risk pathological factors at a total dose of 45–50.4 at 1.8 Gy per daily fraction. Tumor tissue specimens were obtained from all cancer patients, and the adjacent normal non-cancer tissue were obtained from a subset of 30 cancer patients. All these tissues were embedded in paraffin for immunohistochemistry analysis. The survival data were obtained by follow-up records ranged from 7 to 84 months post-surgery (median, 69 months). The OS was defined as the period from the date of surgery until death or the last date of follow-up. The DFS was defined as the period from the date of surgery to recurrence.

In order to measure the level of RIZ1 at mRNA and protein levels, we recruited 20 patients from January to February 2016 following the same protocol. Tumor and their paired normal tissues were collected and stored in liquid nitrogen for analysis.

This study complied with the Helsinki Declaration and was approved by the Ethics Committee of Harbin Medical University (Harbin, China). Informed consents were obtained from all patients or their family members.

### Cell Culture and Transfection

Human cervical cancer HeLa and SiHa cells were obtained from the cell bank of the Committee on Type Culture Collection of the Chinese Academy of Sciences (Shanghai, China). Cells were grown in Dulbecco's Modified Eagle's Medium (DMEM, Invitrogen, Carlsbad, CA, USA) with 10% fetal bovine serum (FBS, Invitrogen). All cells were cultured at 37°C in a humidified atmosphere with 5% CO_2_.

A human cDNA containing RIZ1 coding sequence (NM_012231.4, Invitrogen) was cloned into a pcDNA3.1 vector (Invitrogen) to for cloning the gene (pcDNA3.1-RIZ1-AE). HeLa and SiHa cells were transfected with pcDNA3.1-RIZ1-AE (HeLa-RIZ1-AE and SiHa-RIZ1-AE) or the empty vector pcDNA3.1 (HeLa-vector and SiHa-vector) cells using Lipofectamine 2000 (Invitrogen) according to the manufacturer's instructions. Western blot analysis was performed to determine RIZ1 expression 48 h after transfection.

### Western Blot Analysis

The RIZ1 protein level was analyzed in 20 paired tumor tissues and non-tumor tissues using western blot. Total protein was extracted with RIPA buffer (Invitrogen). Thirty microgram protein was separated on 10% sodium dodecyl sulfate-polyacrylamide gel electrophoresis and transferred to polyvinylidenedifluoride membrane (Millipore Company, Billerica, MA, USA). After blocking with 5% skimmed milk in tris-buffered saline with Tween 20 (TBST), the membrane was incubated with primary antibodies targeting RIZ1 (1:500, cat. AM1194A, Abgent, San Diego, CA, USA) or β-actin (WL0001, Wanlei Bio, Shenyang, China) in TBST containing 1% bovine serum albumin (BSA) overnight at 4°C, followed by incubation with the horseradish peroxidase-labeled secondary antibody (Santa Cruz Biotechnology, Santa Cruz, CA, USA). Protein bands were visualized by an ECL plus chemiluminescence kit (Beyotime Institute of Biotechnology, Haimen, China). β-actin served as a loading control.

### Real-Time PCR

The RIZ1 mRNA level was analyzed in 20 paired tumor tissues and non-tumor tissues using real-time PCR. Total RNA was extracted using Trizol reagent (Life Technologies, Rockville, MD, USA) according to the manufacturer's instructions. One microgram of total RNA was reverse-transcribed to complementary DNA (cDNA) using the PrimeScript 1st Strand cDNA Synthesis Kit (Takara, Dalian, China). Quantitative real-time PCR was conducted by SYBR Green method on a Corbett Rotor-gene 3000 (Corbett Research, Concorde, Australia) real-time thermal cycler. PCR amplification included an initial denaturation at 95°C for 30 s, followed by 45 cycles of denaturation at 95°C for 5 s, annealing at 55°C for 30s, and elongation at 72°C for 30s with detection of double-stranded fluorescence products at the end of each cycle at 85°C. The sequences of the primers are as follows: RIZ1, forward: 5′- TATGTGAATTGGGCTTGC-3′, reverse 5′-GCTGCTATCTCAGGGTTGTC-3′; β-actin (control gene), forward 5′-CTTAGTTGCGTTACACCCTTTCTTG-3′; reverse5′-TGTCACCTTCACCGTTCCAGTTT-3′. The specificity of the PCR was identified and confirmed by melt curve analysis. PCR reactions were performed in triplicate, and data were analyzed through the comparative threshold cycle (C_T_) method.

### Cell Proliferation Assay

Cell proliferation was assessed using MTT assay (WLA021a, Wanlei Bio, Shenyang, China) according to the manufacturer's instructions. Briefly, exponentially growing cells were plated in 96-well plates at 3 × 10^3^ cells per well and incubated for 24, 48, 72, 96, and 120 h. Then, MTT reagent (5 mg/ml) was added in each well and incubated for 4 h at 37°C in a humidified atmosphere with 5% CO_2_. The supernatant was discarded, and the cells were added with DMSO (200 μl). The absorbance at 490 nm was recorded using a microplate reader. Each experiment was repeated at least three times in triplicate.

### Cell Cycle Assay

After culture in six-well plates to a density of 4 × 10^5^ cells/ml for 24 h, cells were harvested, fixed in 70% ice-cold ethanol, and stored at 4°C for 2 h. The fixed cells were washed twice with phosphate-buffered saline (PBS), centrifuged at 2,000 rpm for 5 min, resuspended in PBS, and incubated with 0.2 mg/ml propidium iodide (PI) containing 1 mg/ml RNase A (Sigma) for 30 min in the dark. Flow cytometry analysis was performed using a FACS Calibur cytometer (BD Bioscience, San Jose, CA, USA) to examine cell cycle distribution. The experiments were performed in triplicate.

### Apoptosis Assay

Cell pellets were collected by centrifugation at 1,500 rpm for 5 min, washed with PBS, and resuspended in 500 μl of binding buffer. The pellets were mixed with 5 μl of annexin V-FITC (Thermo Scientific, Rockford, IL, USA) and 10 μl of PI according to the manufacturer's instructions. After incubation at room temperature for 5 min in the dark, the samples were subjected to flow cytometry analysis.

### Wound-Healing Assay

Cell migration was assessed by measuring the movement of cells with a wound-healing assay. Cells were seeded at 4 × 10^5^ cells/well in six-well tissue culture plates and starved overnight in serum-free medium with 1 μg/ml mitomycin C (Sigma). A cell wound area was created and wound healing was quantified as described previously (16). Each experiment was independently performed at least three times.

### Transwell Assays

Transwell Assay was performed on semi-permeable membrane with pore size of 8.0 μm (Costar, Cambridge, MA, USA) to determine cell migration and invasion as described previously (17). At 24 h post-transfection, the cells were seeded at 1 × 10^5^/ml in the upper chamber and cultured in OPTI-MEM (Gibco, USA) without FBS for 24 h. The cells were allowed to migrate in the bottom chamber. Cells that migrate across the membrane was fixed and quantitated by counting in five randomly selected fields under a microscope.

### Immunohistochemical Staining and Evaluation

Formalin-fixed, paraffin-embedded cervical cancer tissues and non-cancer normal tissues were used for RIZ1 immunohistochemical staining. After deparaffinized and blocking, the antigen-antibody reaction was incubated overnight at 4°C. The antigen-antibody complex was visualized with 3,3'-diaminobenzidine reagents. All sections were counterstained with hematoxylin. The primary anti-RIZ1 antibody (cat. AM1194A, Abgent, San Diego, CA, USA) was used at a dilution of 1:50. As a negative staining control, the primary antibody was replaced with PBS.

All immunohistochemistry staining samples were independently estimated by two pathologists in a masked fashion. RIZ1 labeling index was defined as the mean percentage by counting the positive staining of tumor cells for 1,000 cells in three most-labeled areas. As described previously (18), the percentage of positive cells in each case was semi-quantitatively evaluated into one of the following five groups: (1) immunoreactivity completely absent (negative, 0% positive tumor cells); (2) less than 5% positive tumor cells; (3) less than 25% positive tumor cells; (4) less than 50% positive tumor cells; and 5, up to 100% positive tumor cells. Cases with a staining score of 3–5 were defined as “Positive,” and cases with a staining score of 0–2 were defined as “Negative.” In cases of significant disagreement, the scores in question were reviewed by the two original pathologists and a senior pathologist until a consensus was obtained.

### Statistical Analysis

Categorical variables were analyzed using chi-square test. Survival curves were plotted using Kaplan-Meier analysis and evaluated using the log-rank test. Cox proportional hazard regression model was established to identify factors that were independently associated with OS and DFS. In stepwise Cox regression model, we set a significant level with *P* ≤ 0.1 for entering and *P* > 0.1 for removing. Quantitative data are expressed as mean ± SD. Student's *t*-test was used to compare quantitative data population with normal distributions and equal variances. Paired *t*-test was used to compare RIZ1 expression level in cervical cancer tissues with the paired adjacent non-tumor tissues. Statistical analysis was conducted using SPSS Version 16.0 for Windows (SPSS, Chicago, IL, USA). All tests were two-tailed, and differences at *P* < 0.05 were considered statistically significant.

## Results

### RIZ1 Protein Expression Is Down-Regulated in Cervical Cancer

**Figure 1:**
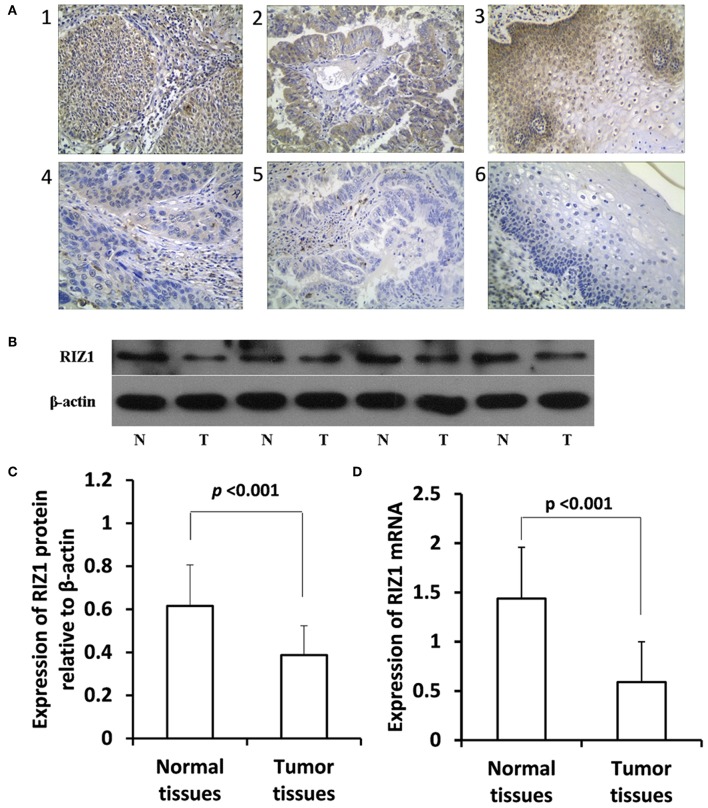
RIZ1 expression was downregulated in cervical cancer tissues compared with the adjacent non-tumor tissues. **(A)** Representative photomicrographs of RIZ1 immunohistochemical staining. Panel 1, positive RIZ1 expression in cervical squamous cell carcinoma; panel 2, positive RIZ1 expression in cervical cervical adenocarcinoma; panel 3, positive RIZ1 expression in normal cervical tissue; panel 4, negative RIZ1 expression in cervical squamous cell carcinoma; panel 5, negative RIZ1 expression in cervical cervical adenocarcinoma; panel 6, negative RIZ1 expression in normal cervical tissue. Original magnification, ×200. **(B)** Total proteins from four pairs of cervical cancer tissues (T) and the adjacent non-tumor tissues (N) were extracted for immunoblotting of RIZ1 with β-actin as a loading control. **(C)** Relative expression RIZ1 to β-actin was quantitated by ImageJ. **(D)** Cervical cancer tissues (T) and the adjacent non-tumor tissues (N) were extracted for quantitative real-time RT-PCR analysis of RIZ1 mRNA with β-actin as an internal control.

The expression of RIZ1 in cervical cancer tissues was assessed by immunohistochemistry. Representative immunohistochemical staining images of RIZ1 in cervical cancer and the paired adjacent non-tumor tissues were shown in Figure 1A. Cells exhibiting brown-yellow particles in the cytoplasm were considered positive for RIZ1 staining. More adjacent non-tumor tissues have positive expression of RIZ1 compared with cancer tissues (28/30, 93.3% vs. 96/268, 35.8% *P* < 0.001). To further confirm this observation, we analyzed protein and mRNA levels of RIZ1 in 20 pairs of tumor and the matched adjacent normal cervical tissues. Consistently, we found significantly decreased protein levels (Figures 1B,C) (*P* < 0.001) and mRNA level of RIZ1 (*P* < 0.001) (Figure 1D) in tumor tissues than the paired non-tumor tissues.

### Relationship Between RIZ1 Expression and the Clinically Pathological Features and Survival of Cervical Cancer Patients

Negative RIZ1 expression was significantly correlated with advanced FIGO stage (*P* = 0.005), deep stromal invasion (*P* = 0.001), lymphovascular space involvement (*P* = 0.041), pelvic lymph node metastasis (*P* = 0.005), and postoperative recurrence (*P* = 0.002). No significant correlation was observed between RIZ1 expression and age (*P* = *0*.166), histological type (*P* = 0.116), histological grade (*P* = 0.168), and tumor size (*P* = 0.269) (Table 1).

**Table 1:** Association analysis between the expression level of RIZ1 and the clinicopathologic factors of FIGO stage I and II cervical cancer patients.

**Variables**	***N* = 268**	**RIZ1 Expression**		***P*-value^**a**^**
		**Negative (%) (*n* = 172)**	**Positive (%) (*n* = 96)**	
**AGE (YEARS)**				
<45	161	98 (57.0)	63 (65.6)	0.166
≥45	107	74 (43.0)	33 (34.4)	
**FIGO STAGE**				
I	115	63 (36.6)	52 (54.2)	**0.005**
II	153	109 (63.4)	44 (45.8)	
**HISTOLOGICAL TYPE**				
SCC	231	144 (83.7)	87 (90.6)	0.116
AC	37	28 (16.3)	9 (9.4)	
**HISTOLOGICAL GRADE**				
G1	70	39 (22.7)	31 (32.3)	0.168
G2	117	76 (44.2)	41 (42.7)	
G3	81	57 (33.1)	24 (25.0)	
**TUMOR SIZE(CM)**				
≤4	190	118 (68.6)	72 (75.0)	0.269
>4	78	54 (31.4)	24 (25.0)	
**DEEP STROMAL INVASION**				
No	91	46 (26.7)	45 (46.9)	**0.001**
Yes	177	126 (73.3)	51 (53.1)	
**LYMPHOVASCULAR SPACE INVOLVEMENT**				
No	189	114 (66.3)	75 (78.1)	**0.041**
Yes	79	58 (33.7)	21 (21.9)	
**PELVIC LYMPH NODE METASTASIS**				
No	187	110 (63.9)	77 (80.2)	**0.005**
Yes	81	62 (36.0)	19 (19.8)	
**POSTOPERATIVE RECURRENCE**				
No	169	97 (56.4)	72 (75.0)	**0.002**
Yes	99	75 (43.6)	24 (25.0)	

*RIZ1, Retinoblastoma protein-interacting zinc-finger gene 1; FIGO, International Federation of Gynecology and Obstetrics; SCC, Squamous cell carcinoma; AC, Adenocarcinoma; Significant P < 0.05 are shown in bold*.

a *χ2 test*.

**Figure 2:**
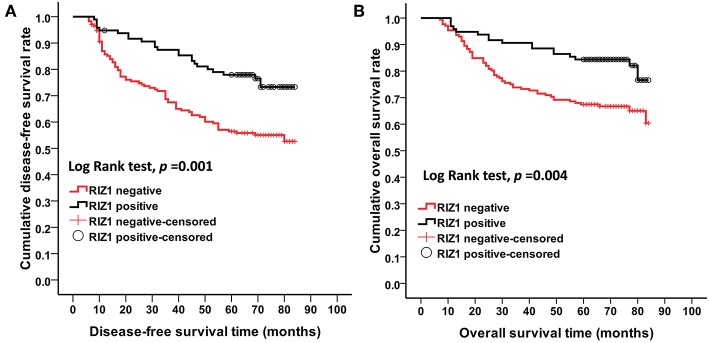
Low RIZ1 expression predicted shorter overall survival (OS) and disease-free survival (DFS) time of cervical cancer patients. Kaplan–Meier curves for the survival of prognosis in 268 patients with FIGO stages I–II cervical cancer according to the categories of negative and positive RIZ1 expression (analyzed with log-rank test). **(A)** Overall survival; **(B)** disease-free survival.

**Figure 3:**
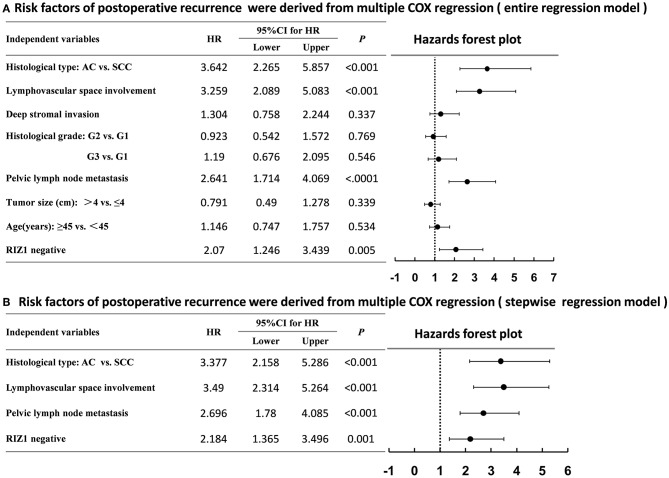
Multiple COX regression analyses of risk factors with the postoperative recurrence in cervical cancer patients using the entire regression model **(A)** and stepwise regression model **(B)**.

**Figure 4:**
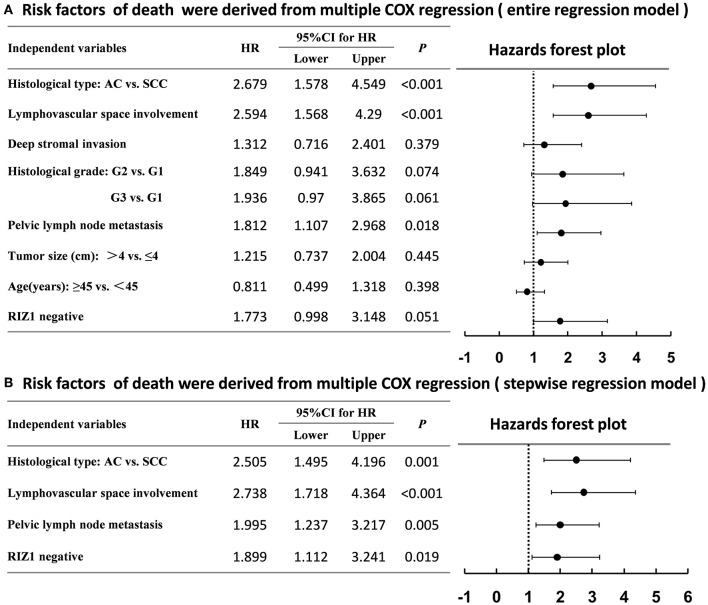
Multiple COX regression analyses of risk factors with the death of cervical cancer patients using the entire regression model **(A)** and stepwise regression model **(B)**.

We then analyzed the correlation of RIZ1 expression with clinical outcome in cervical cancer patients. Univariate and multivariate analyses were conducted to determine the predictors for OS and DFS (Figure 2). The log-rank test showed that patients with positive RIZ1 expression displayed a significantly better DFS (*P* = 0.001) (Figure 2A) and OS (*P* = 0.004) (Figure 2B) compared with patients with negative RIZ1 expression. In addition to RIZ1, multiple COX regression analyses demonstrated that FIGO stage, histological type, histological grade, deep stromal invasion, lymphovascular space involvement, and pelvic lymph node metastasis were significantly correlated with the postoperative recurrence (Figure 3) and death (Figure 4). To adjust the impact of potential confounders, the COX multivariate model (full model and stepwise manner) was used to analyze whether there was a difference in the survival time in patients with negative and positive RIZ1 expression. The results showed that the negative RIZ1 expression was an independent prognostic marker for DFS (HR = 2.184, 95% CI 1.365–3.496, *P* = 0.001, Figure 3) and OS (HR = 1.899, 95% CI 1.112–3.241, *P* = 0.019, Figure 4) after adjustment in stepwise model.

### Overexpression of RIZ1 Inhibits the Proliferation and Induces Cell Cycle Arrest *in vitro*

**Figure 5:**
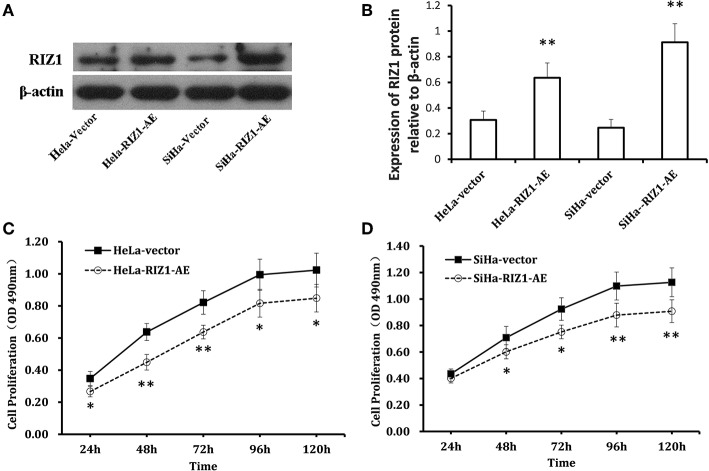
Overexpression of RIZ1 inhibited the proliferation of cervical cancer cells. **(A)** HeLa cells and SiHa cells were transfected with plasmid pcDNA3.1-RIZ1-AE or the empty vector pcDNA3.1. 48 h post-transfection, total proteins from each cell lines were extracted for immunoblotting of RIZ1 with β-actin as a loading control. **(B)** Relative expression RIZ1 to β-actin was quantitated by ImageJ. **(C)** HeLa cells were transfected with plasmid pcDNA3.1-RIZ1-AE or the empty vector pcDNA3.1. The cell viability on day 1 to day 5 after transfection was assessed by MTT assay. **(D)** SeHa cells were transfected with plasmid pcDNA3.1-RIZ1-AE or the empty vector pcDNA3.1. The cell viability on day 1 to day 5 after transfection was assessed by MTT assay. Each point indicates the mean of spectrometric absorbance ± SD of three independent experiments. ^*^P < 0.05, ^**^P < 0.005.

Figures 5A,B show a significant up-regulation of RIZ1 at protein level in HeLa-RIZ1-AE and SiHa-RIZ1-AE compared with HeLa-vector and SiHa-vector, suggesting an effective overexpression of RIZ1 in both cells.

**Figure 6:**
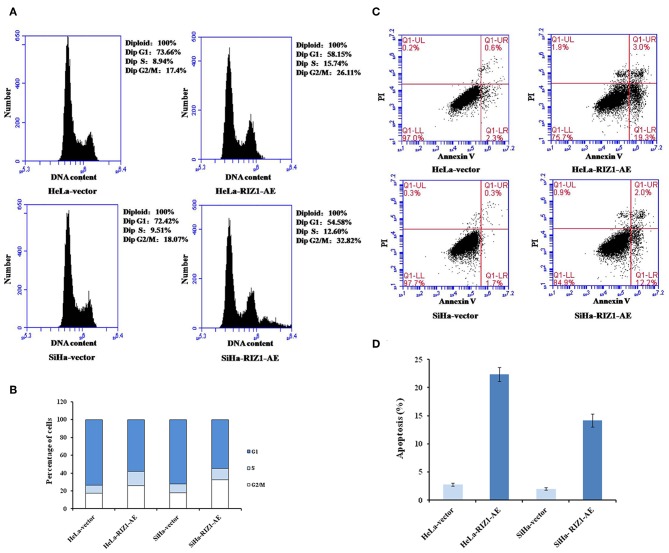
RIZ1 overexpression induced cell cycle arrest at the G2-M phase and apoptosis of cervical cancer cells. HeLa cells and SiHa cells were transfected with plasmid pcDNA3.1-RIZ1-AE or the empty vector pcDNA3.1 for 48 h. **(A)** Cell cycle distribution was assessed with PI staining followed by flow cytometry. **(B)** Percentage of cells at different phases, **(C)** Cell apoptosis was assessed with Annvin V staining followed by flow cytometry, **(D)** Percentage of Annexin V positive cells. The data are the mean ± SD of one representative experiment. Similar results were obtained in three independent experiments.

MTT assay using repeated measurements of variance analysis shows that RIZ1 significantly decreased cell proliferation in HeLa-RIZ1-AE and SiHa-RIZ1-AE cells compared with that in HeLa-vector and SiHa-vector cells (both *P* < 0.05, Figures 5C,D). Flow cytometry revealed that RIZ1 results in significant increase in the percentage of cells at G2-M phase in HeLa-RIZ1-AE and SiHa-RIZ1-AE (Figures 6A,B), suggesting that RIZ1 may inhibit cell proliferation by inducing cell cycle arrest at G2-M phase.

### Overexpression of RIZ1 Induces Apoptosis *in vitro*

An annexin apoptosis assay showed that RIZ1 significantly increased the number of Annexin V positive cells in HeLa-RIZ1-AE and SiHa-RIZ1-AE compared with HeLa-vector and SiHa-vector (both *P* < 0.001, Figures 6C,D), suggesting that overexpression of RIZ1 induces apoptosis in cervical cancer cells.

### Overexpression of RIZ1 Inhibits Migration and Invasion *in vitro*

**Figure 7:**
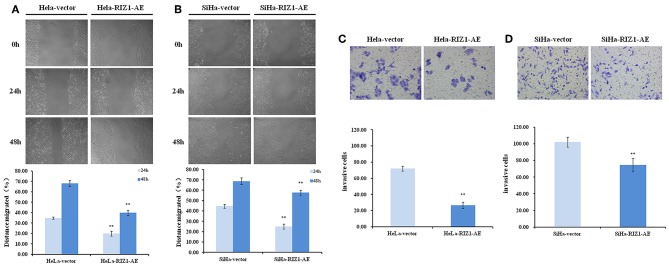
RIZ1 inhibited the migration and invasion of cervical cancer cells *in vitro*. **(A,B)** RIZ1 overexpression reduced the migration capacity of HeLa and SiHa cells as determined by a wound-healing assay, ^**^P < 0.01. **(C,D)** RIZ1 overexpression reduced the invasion capacity of HeLa and SiHa cells was determined by Transwell assays, ^**^P < 0.01.

To explore the potential of RIZ1 in the migration and invasion, a hallmark of cancer cells, we performed *in vitro* wound healing and Matrigel invasion assays. We demonstrated that RIZ1 overexpression led to an apparent suppression of cell migration in HeLa cells (24 h: *P* = 0.001, 48 h: *P* < 0.001; Figure 7A) and SiHa cells (24 h: *P* < 0.001, 48 h: *P* = 0.009; Figure 7B). We also demonstrated that overexpression of RIZ1 markedly reduced the invasive capacity in both HeLa and SiHa cells (Figures 7C,D). The numbers of HeLa-vector and SiHa-vector cells that migrated across both the Matrigel and the insert were 2.7- and 1.4-folds higher than that of HeLa-RIZ1-AE and SiHa-RIZ1-AE cells (72.0 ± 3.0 vs. 26.7 ± 4.0, *P* < 0.001 and 102.0 ± 6.0 vs. 74.3 ± 8.0, *P* = 0.01, respectively). These results showed that overexpression of RIZ1 reduced the migration and invasion of cervical cancer cells *in vitro*.

## Discussion

Despite the improvement in diagnosis, treatment, and prognosis of cancers, cervical cancer remains one of the leading causes of cancer death among women (1). Cervical tumorigenesis is a multistep process starting from benign, cervical intraepithelial neoplasia to *in situ* carcinoma and subsequently to invasive carcinomas. Growing evidence has indicated that genetic, epigenetic and environmental factors contribute together to the multistep development of cervical cancer (19). In precision medicine, it is essential to identify and validate biomarkers that can be used for individual prognosis and treatment in cervical cancer patients (20). RIZ1 is a potential cancer suppressor gene that could serve as a novel biomarker and therapeutic target for various cancers (10, 12–14). RIZ1 contains a conserved positive regulatory domain that methylates histone H3K9, which can repress transcription of various genes. Our previous study has demonstrated that RIZ1 expression level was lower in cervical cancer tissues than that in normal cervical tissues, and the decreased expression was related to the aberrant methylation of the RIZ1 CpG island promoter (15). The current study provided the first evidence on the clinical significance of RIZ1 in the prognosis of cervical cancers.

The study showed that the expression of RIZ1 protein was decreased or nearly lost in cervical cancer tissues at FIGO stages I–II in patients treated with radical hysterectomy. RIZ1 expression was negatively associated with some tumor characteristics, including advanced FIGO stage, lymphovascular space involvement, deep cervical stromal invasion, pelvic lymph node metastasis, and postoperative recurrence. Furthermore, multivariate Cox regression analysis indicated that RIZ1 was an independent prognostic factor for the survival of cervical cancer patients, which was consistent with previous findings (11). Taken together, these results indicated that RIZ1 might be a potent suppressor of cervical cancer, and its expression could serve as a hallmark of patient prognosis in early-stage cervical cancer.

The exact mechanism of RIZ1 in the initiation, development and progression of cancers remains largely unknown. In this study, we showed that ectopic overexpression of RIZ1 significantly inhibited cell proliferation, migration, and invasion, while caused G2-M arrest and induced apoptosis in cervical cancer cell lines. Our finding agrees well with previous reports demonstrating that RIZ1 caused G-2M cell cycle arrest and apoptosis in hepatocellular carcinoma breast cancer, liver cancer, and colon cancer cells (12, 13, 18).

The hypermethylation of CpG islands in gene promoters often leads to transcriptional silencing of genes, including tumor suppressor genes. The RIZ1 promoter possesses the characteristics of a CpG island, suggesting RIZ1 as a target of inactivation through epigenetic mechanisms (21). Our previous study demonstrated that the CpG island of RIZ1 promoter in 37.5% cervical cancer tissues are methylated, while none of normal tissues were methylated (15). The overall decrease of RIZ1 in cervical cancer might result from the transcriptional silencing of RIZ1 by aberrant DNA methylation. Nevertheless, the incidence of RIZ1 gene promoter methylation is not high in other cancers (21–24). Therefore, it would be important to explore the alternative mechanisms of down-regulation of RIZ1 in cervical cancer and other cancer types.

## Conclusion

This study provided evidence that RIZ1 was down-regulated in cervical cancer tissues, and the reduced expression was associated with disease progression. *In vitro* assay shows that RIZ1 overexpression inhibited the proliferation, migration, and invasion, and induced apoptosis. Taken together, down-regulation of RIZ1 expression may contribute to tumor migration and invasiveness, as well as poor survival of cervical cancer patients, suggesting that RIZ1 may be a novel prognostic biomarker for cervical cancer. Further investigations are required to more extensively explore the molecular mechanisms of RIZ1 in cervical cancer.

## Data Availability Statement

The datasets supporting the conclusions of this article are included in this manuscript.

## Ethics Statement

This study complied with the Helsinki Declaration and was approved by the Ethics Committee of Harbin Medical University (Harbin, China). Informed consents were obtained from all patients or their family members.

## Author Contributions

YZ designed the study. SY and TL performed the experiment. HC and ZW wrote and revised the manuscript. YF and SL collected the data. JY polished the language. All authors reviewed and approved the final version.

### Conflict of Interest

The authors declare that the research was conducted in the absence of any commercial or financial relationships that could be construed as a potential conflict of interest.
